# Characterization of Adrenal miRNA-Based Dysregulations in Cushing’s Syndrome

**DOI:** 10.3390/ijms23147676

**Published:** 2022-07-12

**Authors:** Sharmilee Vetrivel, Ru Zhang, Mareen Engel, Andrea Oßwald, Deepika Watts, Alon Chen, Ben Wielockx, Silviu Sbiera, Martin Reincke, Anna Riester

**Affiliations:** 1 Medizinische Klinik und Poliklinik IV, LMU Klinikum, Ludwig-Maximilians-University, 80336 Munich, Germany; sharmilee.vetrivel@med.uni-muenchen.de (S.V.); ru.zhang@med.uni-muenchen.de (R.Z.); andrea.osswald@med.uni-muenchen.de (A.O.); martin.reincke@med.uni-muenchen.de (M.R.); 2 Department of Stress Neurobiology and Neurogenetics, Max Planck Institute of Psychiatry, 80804 Munich, Germany; m_engel@protonmail.com (M.E.); alon.chen@weizmann.ac.il (A.C.); 3 Institute of Clinical Chemistry and Laboratory Medicine, Technische Universität Dresden, 01307 Dresden, Germany; deepika.watts@uniklinikum-dresden.de (D.W.); ben.wielockx@tu-dresden.de (B.W.); 4 Department of Neurobiology, Weizmann Institute of Science, Rehovot 76100, Israel; 5 Department of Internal Medicine I, Division of Endocrinology and Diabetes, University Hospital, University of Würzburg, 97080 Würzburg, Germany; sbiera_s@ukw.de

**Keywords:** cortisol, ACTH, miRNA, Cushing’s, hypercortisolism, pituitary

## Abstract

MiRNAs are important epigenetic players with tissue- and disease-specific effects. In this study, our aim was to investigate the putative differential expression of miRNAs in adrenal tissues from different forms of Cushing’s syndrome (CS). For this, miRNA-based next-generation sequencing was performed in adrenal tissues taken from patients with ACTH-independent cortisol-producing adrenocortical adenomas (CPA), from patients with ACTH-dependent pituitary Cushing’s disease (CD) after bilateral adrenalectomy, and from control subjects. A confirmatory QPCR was also performed in adrenals from patients with other CS subtypes, such as primary bilateral macronodular hyperplasia and ectopic CS. Sequencing revealed significant differences in the miRNA profiles of CD and CPA. QPCR revealed the upregulated expression of miR-1247-5p in CPA and PBMAH (log2 fold change > 2.5, *p* < 0.05). MiR-379-5p was found to be upregulated in PBMAH and CD (log2 fold change > 1.8, *p* < 0.05). Analyses of miR-1247-5p and miR-379-5p expression in the adrenals of mice which had been exposed to short-term ACTH stimulation showed no influence on the adrenal miRNA expression profiles. For miRNA-specific target prediction, RNA-seq data from the adrenals of CPA, PBMAH, and control samples were analyzed with different bioinformatic platforms. The analyses revealed that both miR-1247-5p and miR-379-5p target specific genes in the WNT signaling pathway. In conclusion, this study identified distinct adrenal miRNAs as being associated with CS subtypes.

## 1. Introduction

Cushing’s syndrome (CS) results from the excessive secretion of cortisol, leading to visceral obesity, resistance to insulin, osteoporosis, and altered lipid and glucose metabolism [1,2]. Excessive production of cortisol by the adrenal glands can be either ACTH-dependent or -independent. In the majority of patients, hypercortisolism is due to ACTH secretion by corticotroph adenomas of the pituitary gland (Cushing’s disease, CD) or by ectopic tumors [3]. Approximately 20% of cases are ACTH-independent, where cortisol is secreted autonomously by the adrenal cortex. The pathology of ACTH-independent cases is diverse; they are most often caused by unilateral cortisol-producing adrenocortical adenomas (CPA). Rare causes are cortisol-secreting adrenocortical carcinomas (ACC), primary bilateral macronodular adrenocortical hyperplasia (PBMAH), bilateral CPAs, and primary pigmented nodular adrenal disease (PPNAD) [4,5]. Irrespective of the subtype, prolonged exposure to cortisol in CS is associated with increased mortality and cardiovascular morbidity in its patients [6]. Treatment is based on the underlying cause of hypercortisolism, with pituitary surgery or adrenalectomy being the preferred choice. Medical therapy options in CS are few and consist of pituitary-directed drugs, steroid synthesis inhibitors, and glucocorticoid receptor antagonists [7]. For the timely diagnosis and targeted management of CS and its subtypes, a comprehensive understanding of cortisol secretion, in terms of canonical signaling pathways as well as upstream epigenetic factors, is needed.

MiRNA molecules have emerged as key epigenetic players in the transcriptional regulation of cortisol production. Briefly, the deletion of *Dicer* in adrenals, a key miRNA processing enzyme, revealed diverse expression changes in miRNAs along with related changes in steroidogenic enzymes such as *Cyp11b1* [8]. Furthermore, key enzymes in the cortisol biosynthesis pathway, namely *Cyp11a1*, *Cyp21a1*, *Cyp17a1*, *Cyp11b1*, and *Cyp11b2*, were also found to be regulated by various miRNAs (miRNA-24, miRNA-125a-5p, miRNA-125b-5p, and miRNA-320a-3p) in in vitro studies [9]. Consequently, various studies have also characterized miRNA expression profiles in CS subtypes. Importantly, miRNA expression in the corticotropinomas of CD patients was found to vary according to USP8 mutation status [10]. Other studies have also identified specific miRNA candidates and associated target genes in the adrenals of patients with PPNAD [11], PBMAH [12,13], and massive macronodular adrenocortical disease [14]. Interestingly, no common miRNA candidates were found among these studies, indicating the specificity of miRNAs to the different underlying pathologies in CS.

There are limited studies directly comparing miRNA expression profiles of ACTH-dependent and ACTH-independent CS patients. Consequently, in our previous study, we found differences in expression profiles when comparing circulating miRNAs in CD and CPA patients [15]. We hypothesized that the presence of ACTH possibly influences the miRNA profile in serum due to the upstream differential expression in the origin tissues. In this study, we aim to further explore this hypothesis by comparing the miRNA expression profile of adrenal tissues in ACTH-dependent and ACTH-independent CS. In brief, miRNA specific sequencing was performed in two prevalent subtypes of CS: in CD, the most prevalent ACTH-dependent form; and in CPA, the most prevalent ACTH-independent form. Specific miRNA candidates related to each subtype were further validated in other forms of CS. To further investigate our hypothesis, the response of miRNA candidates following ACTH stimulation was assessed in mice, and the expression of miRNAs in murine adrenals was subsequently investigated. Finally, an extensive targeted gene analysis was performed based on in silico predictions, RNA-seq data, and luciferase assays.

## 2. Results

### 2.1. Differentially Expressed miRNAs

**Figure 1:**
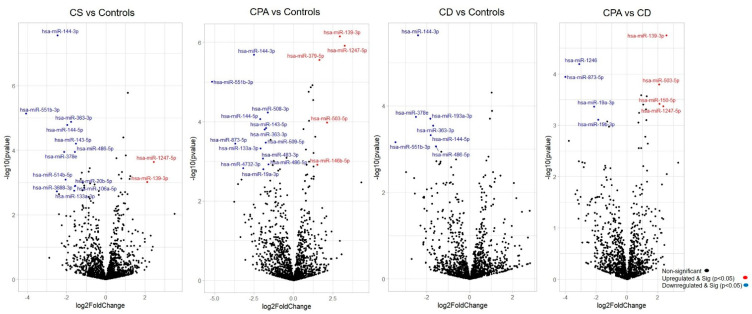
Differentially expressed miRNAs from sequencing. Volcano plot showing the relationship between fold change (log2 fold change) and statistical significance (−log10 *p* value). The red points in the plot represent significantly upregulated miRNAs, while blue points represent significantly downregulated miRNAs. CPA, cortisol producing adenoma; CD, Cushing’s disease; Cushing’s syndrome represents CPA and CD, taken together.

**Figure 2:**
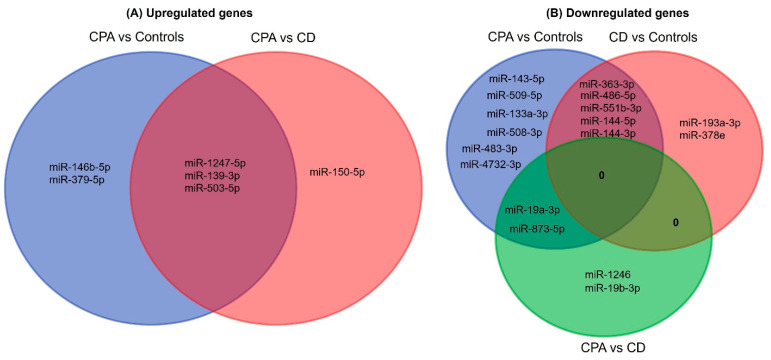
Venn analyses of the common significant miRNAs from each group. The significantly expressed miRNAs from each sequencing analysis were shortlisted and compared between the groups. CPA, cortisol producing adenoma; CD, Cushing’s disease.

NGS revealed differentially expressed miRNAs between the different groups analyzed (Figure 1). CD and CPA taken together as CS showed a differentially expressed profile (42 significant miRNAs) in comparison to controls. Moreover, individually, CPA and CD were found to show a significantly different expression profile in comparison to controls (n = 38 and n = 17 miRNAs, respectively). Interestingly, there were no significantly upregulated genes in the adrenals of patients with CD in comparison to the control adrenals. A comparative analysis of the top significant miRNAs (log2 fold change (log2 FC) > 1.25 & *p* < 0.005) between the two groups was performed and the representative Venn diagrams are given in Figure 2. Briefly, miR-1247-5p, miR-139-3p, and miR-503-5p were significantly upregulated in CPA, in comparison to both CD and controls. Furthermore, miR-150-5p was specifically upregulated in CPA as compared to CD. Several miRNAs (miR-486-5p, miR-551b-3p, miR-144-5p, miR-144-3p, and miR-363-3p) were found to be significantly downregulated in the groups of CPA and CD in comparison to controls. MiR-19a-3p and miR-873-5p were found to be commonly downregulated in CPA in comparison to both CD and controls. Principal component analyses based on miRNA sequencing did not identify any major clusters among the samples. Furthermore, the miRNA profile was not different among the CPA samples based on the mutation status of PRKACA (Supplementary Materials Figure S1).

### 2.2. Validation and Selection of Candidate miRNAs

For validation by QPCR, the most significant differentially expressed miRNAs (log2 FC > 1.25 & *p* < 0.005) among the groups were chosen (Table S1). According to the current knowledge, upregulated miRNAs are known to contribute more to pathology than downregulated miRNAs [16]. Since the total number of significantly upregulated miRNAs was six, all these miRNAs were chosen for validation. Contrarily, 25 miRNAs were significantly downregulated among the groups. In particular, miR-486-5p, miR-551b-3p, miR-144-5p, miR-144-3p, and miR-363-3p were found to be commonly downregulated in the CS group in comparison to controls; therefore, these miRNAs were chosen for validation.

**Figure 3:**
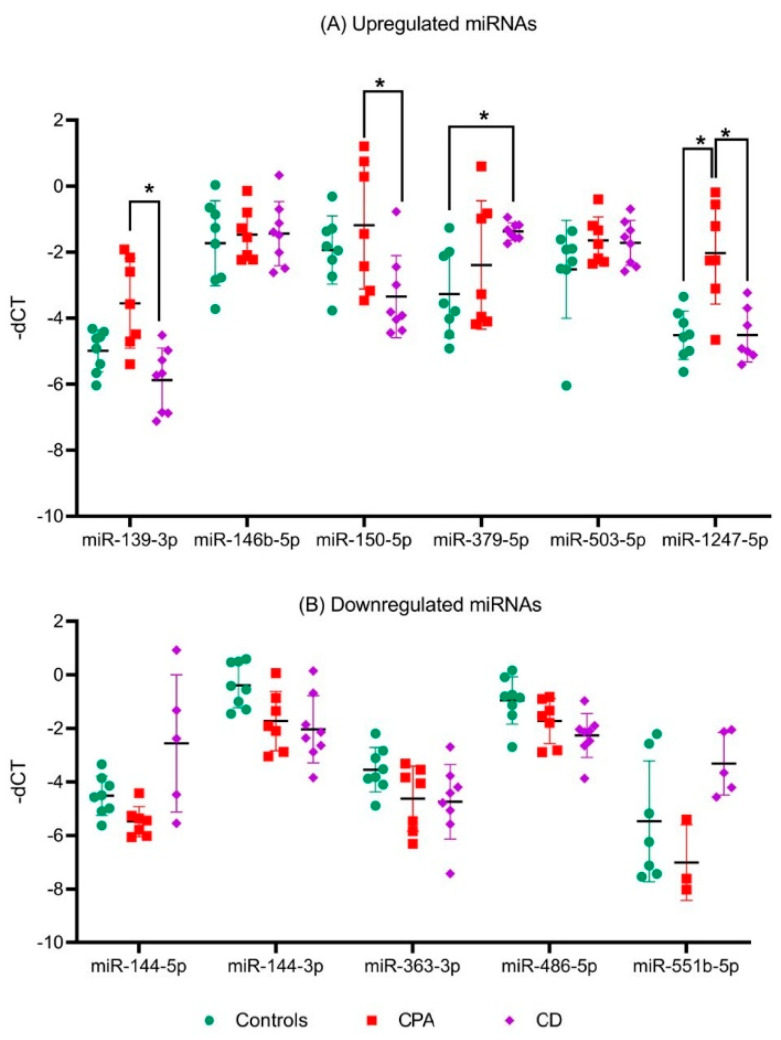
QPCR analyses of significant miRNAs from sequencing analyses. Data are represented as mean ± standard deviation (SD) of −dCT values: (**A**) Expression analysis of significantly upregulated miRNAs; (**B**) Expression analysis of common significantly downregulated miRNAs. Housekeeping gene: miR-16-5p. Statistics: ANOVA test with Bonferroni correction to detect significant differences between patient groups with at least a significance of *p*-value < 0.05 (*).

**Figure 4:**
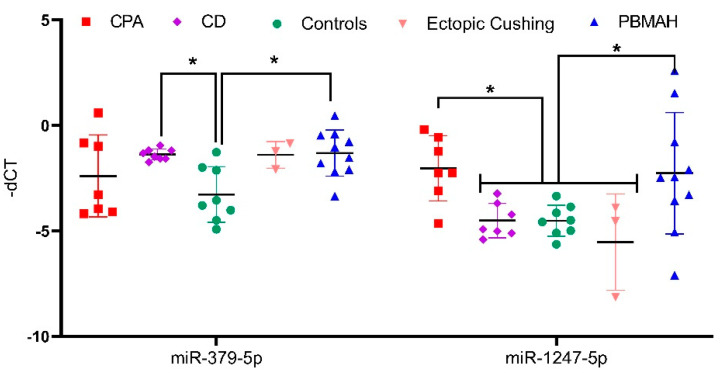
QPCR analyses of significantly upregulated miRNAs from validation QPCR. Data are represented as mean ± standard deviation (SD) of −dCT values. Housekeeping gene: miR-16-5p. Statistics: ANOVA test with Bonferroni correction to detect significant differences between patient groups with at least a significance of *p*-value < 0.05 (*).

Among the upregulated miRNA candidates, miR-1247-5p QPCR expression confirmed the NGS data (Figure 3A, Table S1). Moreover, miR-150-5p and miR-139-3p were upregulated in CPA specifically in comparison to CD, and miR-379-5p was upregulated in CD in comparison to controls by QPCR. In the case of downregulated genes, none of the selected miRNAs could be confirmed by QPCR (Figure 3B). Thus, analysis of the six upregulated and five downregulated miRNAs from NGS yielded two significantly upregulated miRNA candidates, miR-1247-5p in CPA and miR-379-5p in CD, when compared to controls. These miRNA candidates were taken up for further QPCR validation in an independent cohort of other subtypes of CS (Figure 4), namely ACTH-dependent ectopic CS (n = 3) and ACTH-independent PBMAH (n = 10). The QPCR analysis in the other subtypes revealed miR-1247-5p to be consistently upregulated in ACTH-independent CS (PBMAH and CPA) in comparison to ACTH-dependent CS (CD and ectopic CS) and controls. On the other hand, miR-379-5p was upregulated in CD and PBMAH in comparison to controls.

### 2.3. In Vivo Assessment of ACTH-Independent miR-1247-5p

**Figure 5:**
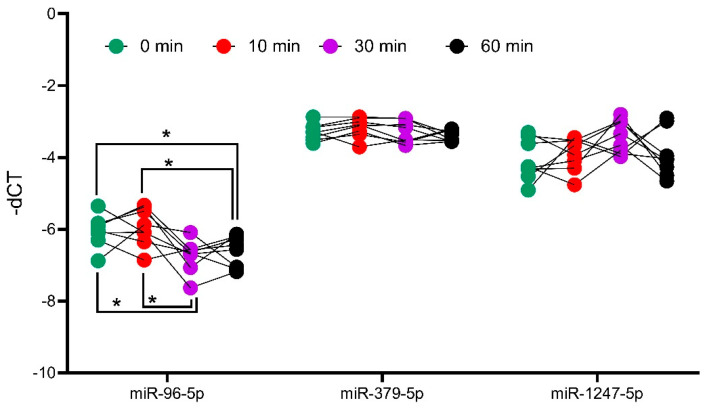
Analysis of miRNA expression in ACTH stimulated mice tissue. QPCR analyses of positive controls, miR-96-5p, and candidates miR-379-5p and miR-1247-5p. Mice were injected with ACTH, and adrenals were collected at different timepoints to assess the impact of ACTH on miRNA expression. Data are represented as mean ± standard deviation (SD) of −dCT values. Housekeeping gene: miR-26a-5p. Statistics: ANOVA test with Bonferroni correction to detect significant differences between patient groups with at least a significance of *p*-value < 0.05 (*).

To analyze the influence of ACTH on miRNA expression, the expression of miR-1247-5p and miR-379-5p were assessed in the adrenal tissues of ACTH stimulated mice at different time points. For this analysis, miR-96-5p was taken as a positive control, as it has previously been reported to be differentially expressed in ACTH stimulated mice [17]. The analyses revealed that the expression of miR-1247-5p and miR-379-5p did not change at different timepoints of the ACTH stimulation (Figure 5). Meanwhile, the positive control of mir-96-5p showed a dynamic expression pattern with upregulation after 10 min, followed by downregulation at the subsequent 30 and 60 min time points, in concordance with previously reported findings [18].

### 2.4. In Silico Analyses of miRNA Targets

**Figure 6:**
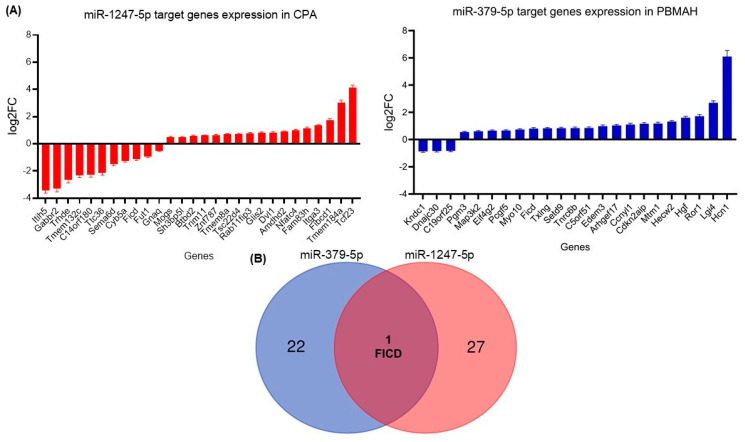
(**A**) Differentially expressed target genes of miRNAs from sequencing. Data are represented as log2 fold change in comparison to the controls. Statistics: ANOVA test with Bonferroni correction to detect significant differences between patient groups with at least a significance of *p*-value < 0.05. (**B**) Venn analyses of common significant miRNA target genes and related pathways. The significantly expressed targets from each sequencing analysis were shortlisted and compared between the groups. Predicted pathways of the targets from the Panther database were shortlisted and compared between the groups.

Two diverse approaches were employed for a comprehensive in silico analysis of the miRNA targets. First, the predicted targets of miR-1247-5p and miR-379-5p were taken from the TargetScan database, which identified miRNA–mRNA target pairs based on sequence analyses [19]. The expression status of these targets was then checked in the RNA sequencing data from CPA vs. controls (miR-1247-5p) and PBMAH vs. controls (miR-379-5p). Targets that showed significant expression changes in the sequencing data were shortlisted (Figure 6A). Among the 1061 predicted miR-1247-5p targets, 28 genes were found to show significant expression changes in CPA (20 upregulated, 8 downregulated). On the other hand, for 124 predicted miR-379-5p targets, 23 genes were found to show significant expression changes in PBMAH (20 upregulated, 3 downregulated). Interestingly, the selected targets were found to be unique for each miRNA, except for FICD (FIC domain protein adenylyltransferase) (Figure 6B).

### 2.5. In Vitro Analyses of miR-1247-5p Targets

**Figure 7:**
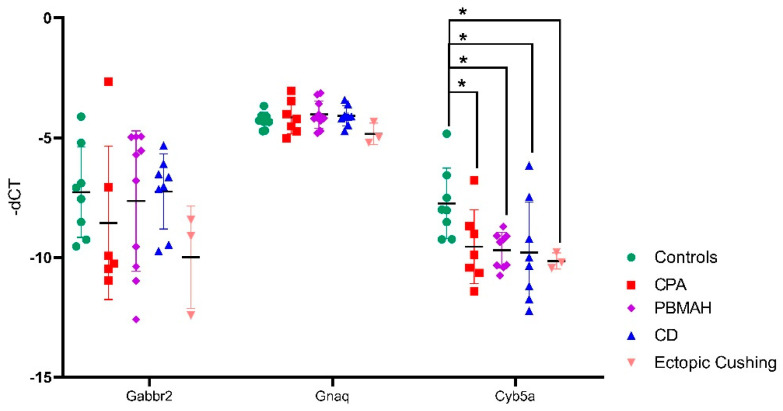
QPCR analyses of the top predicted targets of miR-1247-5p. Data are represented as mean ± standard deviation (SD) of −dCT values. Housekeeping gene: PPIA. Statistics: ANOVA test with Bonferroni correction to detect significant differences between patient groups with at least a significance of *p*-value < 0.05 (*).

**Figure 8:**
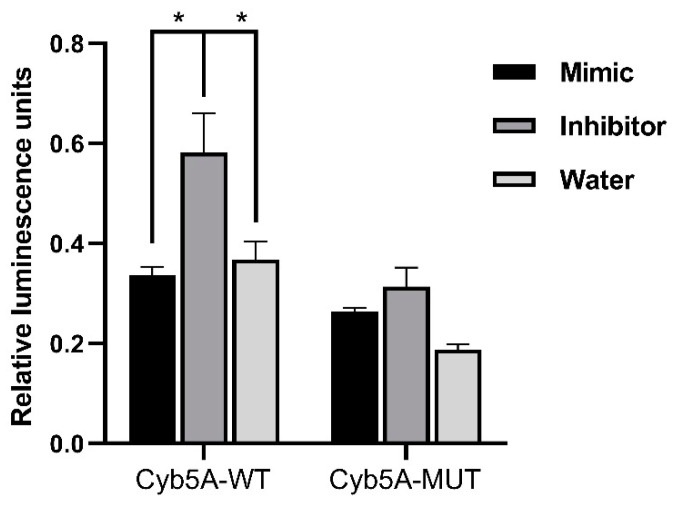
Results of dual luminescence assay on cells transfected with miR-1247-5p mimics and related controls. Cells were transfected with plasmids containing either the WT or Mut miRNA binding sequence in Cyb5a. Either miR-1247-5p mimics or miR-1247-5p inhibitors were transfected together with the respective plasmids. Data are represented as mean ± standard error of mean (SEM) of relative luminescence unit values. Statistics: ANOVA test with Bonferroni correction to detect significant differences between patient groups with at least a significance of *p* value < 0.05 (*).

For in vitro analyses, we focused on downregulated targets, as we expect our upregulated miRNA candidates to cause a downregulation of the target mRNAs. For our downregulated mRNAs, only targets of miR-1247-5p were found to have published links to CS, namely Cyb5a, Gabbr2, and Gnaq (Table 1). Therefore, these three targets were then verified by QPCR in the groups of CPA, CD, PBMAH, ectopic CS, and controls (Figure 6). Only Cyb5A was found to be significantly downregulated in ACTH-dependent forms (ectopic CS and CD) as well as in ACTH-independent CS (PBMAH and CPA) in comparison to controls. Consequently, to assess whether Cyb5a is indeed regulated by miR-1247-5p, a dual luciferase assay was performed. To prove our hypothesis, treatment of Cyb5a-WT cells with miR-1247-5p mimic was expected to lead to a reduced luminescence, whereas no effects were expected in cells treated with the miR-1247-5p inhibitor or the Cyb5a-mutant (with a mutation in the miR-1247-5p binding site). As shown in Figure 7, transfection of miR-1247-5p significantly reduced luminescence from Cyb5a-WT in comparison to cells transfected with Cyb5a-WT and miR-1247-5p inhibitors. However, these predicted binding results were not found to be specific, as there were no significant differences when compared to wells transfected with Cyb5a-WT alone (Figure 8). Consecutively, when the mutated Cyb5a-Mut were transfected along with the mimics and inhibitors, no significant differences in luminescence were observed in the transfected population. Thus, direct interaction between miR-1247-5p and its predicted target gene Cyb5A could not be conclusively proven using this luciferase assay.

### 2.6. Pathway Analyses of miRNA Targets

**Figure 9:**
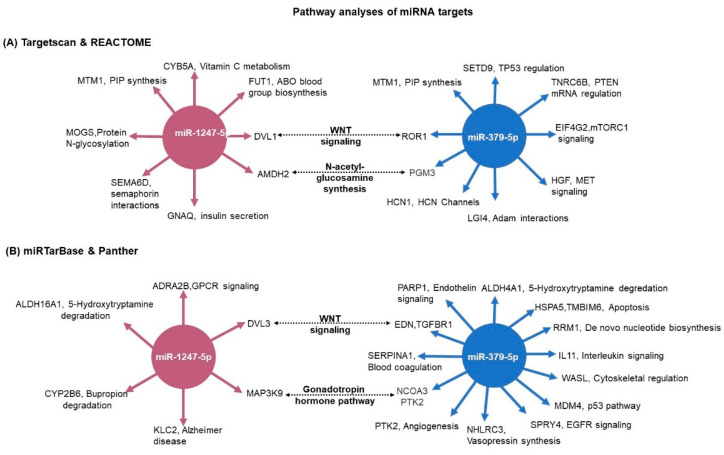
Pathway analyses of miRNA target genes. (**A**) The predicted targets were matched with the RNA-seq expression data. For miR-1247-5p, CPA vs. controls expression data; and for miR-379-5p, PBMAH vs. controls expression data. The significantly expressed target genes were then subjected to pathway analyses by Reactome. The significantly enriched pathway networks (*p* < 0.05) and their related genes are given. (**B**) The experimentally validated target genes from miRTarBase were analyzed for their role in pathways by the Panther database. The target genes and their related pathways are given. The commonly represented pathways are marked in bold.

For the pathway analysis (Reactome) we used the 28 predicted miRNA-1247-5p targets and the 23 predicted miRNA-379-5p targets from TargetScan, which were significantly differently expressed in the RNA-seq (Figure 6). Concurrently, the pathways commonly enriched by both miRNAs included the WNT signaling pathway and N-acetyl-glucosamine synthesis (Figure 9A). As a complementary approach, in silico analyses were also performed based on the targets from miRTarBase. In this database, targets are shortlisted based on published experimental results. In this analysis, miR-1247-5p (n = 21) and miR-379-5p targets (n = 85) were unique. While the validated targets of miR-379-5p were found to show significant changes in expression in the RNA-seq data from PBMAH (n = 12), none of the validated miR-1247-5p targets were found to show significant expression changes in the RNA-seq data from CPA. Therefore, all the validated targets of the miRNAs were subjected to pathway analyses (Panther). Interestingly, the WNT signaling pathway was also found to be commonly regulated by both miRNAs using this approach (Figure 9B). Finally, the expression status of target genes related to WNT signaling pathways were checked in our RNA-seq data (Figure S2). Given the upregulated status of the miRNAs, a downregulated expression of its target genes was expected. However, a significantly upregulated expression was observed for DVL1 in CPA in comparison to controls and for ROR1 in PBMAH in comparison to controls.

## 3. Discussion

MiRNAs are fine regulators of both physiology and pathology and have diverse roles as diagnostic biomarkers as well as therapeutic targets. While circulating miRNAs have been investigated as potential biomarkers for hypercortisolism in CS subtypes (36), comprehensive analyses of their pathological role in CS subtypes have not yet been performed. This study hoped to uncover the pathological role of miRNAs in different CS subtypes as well as identify unique epigenetic targets contributing to hypercortisolism in these subtypes. As such, miRNA sequencing was performed in the ACTH-independent CPA and ACTH-dependent CD, with additional QPCR validation in PBMAH and ectopic CS. As expected, miRNA expression profiles in CD and CPA were very different.

### 3.1. ACTH-Independent Upregulated miRNAs in CS

Among the analyzed miRNAs, only miR-1247-5p and miR-379-5p showed the most prominent changes in expression levels. Briefly, miR-1247-5p was significantly upregulated in ACTH-independent forms of CS, CPA, and PBMAH (Figure 1 and Figure 3) while miR-379-5p was found to be upregulated in CD and PBMAH, in comparison to controls. Even though CD and PBMAH represent CS subtypes with different ACTH dependence, albeit both with hyperplastic tissue, it is interesting to find a shared miRNA expression status. Concurrently, miRNAs have been identified as dynamic players in regulating the acute effect of ACTH on adrenal steroidogenesis in in vivo murine studies [20,21]. Further diverse miRNAs have been characterized to regulate steroidogenesis in ACTH and dexamethasone treated rats [22] (suppressed ACTH) as well as in in vitro studies [20]. It is possible that miR-379-5p contributes to the adrenal hyperplasia present in both PBMAH and CD by targeting specific genes related to hyperplasia, and miR-1247-5p by contributing to cortisol production independent of ACTH regulation in CPA and PBMAH.

Interestingly, the miRNA candidates (mir-1247-5p and miR-379-5p) in our study have not been previously characterized in any of these studies. Furthermore, the expression of mir-1247-5p and miR-379-5p were found to be independent of ACTH stimulation, underlying their role in CS independently of the HPA axis control and postulating specific regulatory processes.

### 3.2. Target Genes of miRNAs in CS

Initially, we focused on the selection of known CS specific target genes that could be directly repressed by miRNAs, thereby contributing to pathology. The predicted target genes of miR-1247-5p and miR-379-5p were assessed for their downregulated expression status in the RNA-seq data. Among the selected target genes, only Cyb5a was found to be significantly downregulated in all forms of CS (Figure 6). Cytochrome b5 (CYB5A) is a marker of the zona reticularis and is an important regulator of androstenedione production [23,24]. Based on its role in adrenal steroidogenesis, it is possible that Cyb5a is downregulated by miR1247-5p. To prove our hypothesis, a dual luciferase assay was performed in HELA cell line to identify a direct interaction between Cyb5a and miR-1247-5p (Figure 7). Unfortunately, a direct interaction could not be proven, indicating that miR-1247-5p perhaps regulates its target genes in different ways.

### 3.3. Pathway Analyses of miRNA Targets

To identify miRNA specific regulatory processes, comprehensive target and pathway analyses were performed. Independent pathway analyses of the respective targets with two different databases of Reactome and Panther showed the WNT signaling pathway as a common targeted pathway of both mir-1247-5p and miR-379-5p (Figure 8). The WNT signaling pathway represents a crucial regulator in diverse developmental as well as pathological processes with tissue-specific effects [25,26]. Consequently, the WNT pathway has been largely characterized as one of the dysregulated pathophysiological mechanisms in CPA. Mutations in PRKACA, one of the WNT signaling proteins, are present in approximately 40% of CPA [27]. In the case of CD, dysregulated WNT signaling has been characterized as promoting proliferation in ACTH-secreting pituitary adenomas [28]. Moreover, activating mutations in beta catenin, one of the WNT signaling pathways, has been characterized as driving adrenal hyperplasia through both proliferation-dependent and -independent mechanisms [29]. Thus, it could be hypothesized that by targeting specific genes in the pathway, miRNAs drive specific pathophysiological processes in diverse CS subtypes.

### 3.4. MiRNA Target Genes in WNT Signaling

DVL1 (TargetScan) and DVL3 (miRTar) are the shortlisted target genes of miR-1247-5p in the WNT signaling pathway. These genes are members of canonical WNT pathways and, importantly, activation of the cytoplasmic effector Dishevelled (Dvl) is a critical step in WNT/β-catenin signaling initiation [30,31]. Interestingly, no difference in DVL1 and DVL3 gene expression was found in the analyses of ACTH-secreting pituitary adenomas [32]. Therefore, it could be possible that DVL1 and DVL3 are only targeted by miR-1247-5p specifically in the adrenal of CPA and PBMAH patients, leading to its characterized tumor progression. EDN1, TGFBR1 (TargetScan), and ROR1 (miRTar) were the target genes of miR-379-5p related to the WNT pathway. In epithelial ovarian cancer, Endothelin-1 (EDN-1) was found to regulate the epithelial–mesenchymal transition (EMT) and a chemoresistant phenotype [33]. In the case of receptor tyrosine kinase-like orphan receptor 1 (ROR1), higher expression of the gene was associated with a poor prognosis in ovarian cancer [34]. Concurrently, suppression of TGFBR1-mediated signaling by conditional knockout in mice was found to drive the pathogenesis of endometrial hyperplasia, independent of the influence of ovarian hormones [35]. Therefore, it could be hypothesized that the dysregulated expression of these factors in adrenals could trigger similar hyperplastic effects mediated by these factors, as in ovarian tissues.

### 3.5. Bottlenecks and Future Outlook

Interestingly, among these genes, only DVL1 and ROR1 were found to be significantly upregulated in the RNA-seq data (Figure S2). The major regulatory role of miRNAs in gene expression come from their ability to repress gene expression at the level of transcription and translation. There are also reports of miRNA-mediated gene upregulation; however, the physiological evidence of the role is still unresolved [36]. Therefore, it is interesting to see the selected targets of miR-1247-5p and miR-379-5p upregulated. Moreover, it should be noted that most of the experimentally validated miRNA targets were identified by CLIP methods [37]. Crosslinking immunoprecipitation (CLIP) are binding assays that provide genome-wide maps of potential miRNA-target gene interactions based on sequencing. Moreover, these assays do not make functional predictions on the outcome of miRNA binding, and neither do upregulation or downregulation [38,39]. Therefore, in our current experimental setting, we could only identify potential miRNA target genes and speculate on their pathological role based on the published literature and in silico analyses. Furthermore, extensive mechanistic analyses based on these potential targets could help in elaborating the specific epigenetic pathways that fine-tune CS pathology in different subtypes.

## 4. Materials and Methods

### 4.1. Sample Collection and Ethics Approval

All patients were registered in the German Cushing’s Registry, the ENS@T or/and NeoExNET databases (project number protocol code 379-10 and 152-10). The study was approved by the Ethics Committee of the University of Munich. All experiments were performed according to relevant guidelines and protocols, and written informed consent was obtained from all patients involved. The adrenal samples used in the sequencing (miRNA and RNA) belong to the same patient.

For miRNA-specific next-generation sequencing (NGS), a total of 19 adrenocortical tissue samples were used. The cohort consisted of the following patient groups: ACTH-independent CPA, n = 7; ACTH-dependent hypertrophic adrenals of CD patients after bilateral adrenalectomy, n = 8; normal adjacent adrenal tissue from patients with pheochromocytoma as controls, n = 8. For QPCR validation, the patient groups included adrenal tissue from ACTH-independent PBMAH, n = 10, and ACTH-dependent ectopic CS, n = 3.

In the case of mRNA sequencing, a total of 23 adrenocortical tissue samples were used. This includes the following patient groups: CPA, n = 7; PBMAH, n = 8; normal adjacent adrenal tissue from patients with pheochromocytoma as controls, n = 8.

The clinical characteristics of the patients are given in Table 2. Furthermore, of the eight CPA samples in the study, three of them carried known somatic driver mutations in the PRKACA gene and in the ten PBMAH samples, two carried germline mutations in the ARMC5 gene.

The adrenal tissues were stored at −80 °C. Total RNA isolation was carried out from all adrenal cortex samples by an RNeasy Tissue Kit (Qiagen, Hilden, Germany). The isolated RNA was kept frozen at −80 °C until further use.

### 4.2. MiRNA and RNA Sequencing

RNA integrity and the absence of contaminating DNA were confirmed by Bioanalyzer RNA Nano (Agilent Technologies, Santa Clara, CA, USA) and by Qubit DNA High sensitivity kits, respectively. Sequencing libraries were prepared using the Illumina TruSeq Small RNA Library Preparation Kit. NGS was performed on 2 lanes of an Illumina HiSeq2500 (Illumina, CA, USA) multiplexing all samples (paired end read, 50 bp). The quality of sequencing reads was verified using FastQC0.11.5 (http://www.bioinformatics.babraham.ac.uk/projects/fastqc, date last accessed: 13 March 2020) before and after trimming. Adapters were trimmed using cutadapt [40]. Reads with <15 bp and >40 bp insert sequences were discarded. An alignment of reads was performed using miRBase V21 [41,42] with sRNAbench [43]. EdgeR and DeSeq in R were used for further analyses [44,45]. MiRNAs with at least 5 raw counts per library were included. RNA-seq was performed by Qiagen, Hilden, Germany. For mRNA, sequencing was performed on Illumina NextSeq (single end read, 75 bp). Adapter and quality trimming were performed by the “Trim Reads” tool from CLC Genomics Workbench. Furthermore, reads were trimmed based on quality scores. The QC reports were generated by the “QC for Sequencing Reads” tool from CLC Genomics Workbench. Read mapping and gene quantification were performed by the “RNA-seq Analysis” tool from CLC Genomics Workbench [46]. The miRNA-seq data generated in this study have been submitted to the NCBI (PRJNA847385).

### 4.3. Validation of Individual miRNAs

MiRNAs and genes significantly differentially expressed by NGS were validated by QPCR. Reverse transcription of miRNA-specific cDNA was performed by using the TaqMan MicroRNA Reverse Transcription Kit (Thermo Fisher Scientific, Munich, Germany), and the reverse transcription of RNA genes was done by using the Superscript VILO cDNA synthesis Kit (Thermo Fisher Scientific, Munich, Germany). 50 ng of RNA was used for each of the reverse transcription reactions. Quantitative real-time PCR was performed using the TaqMan Fast Universal PCR Master Mix (2×) (Thermo Fisher Scientific, Munich, Germany) on a Quantstudio 7 Flex Real-Time PCR System (Thermo Fisher Scientific, Munich, Germany) in accordance with the manufacturer’s protocol. All QPCR reactions were performed in a final reaction volume of 20 μL and with 1 μL of 1:5 diluted cDNA. Negative control reactions contained no cDNA templates. Gene expression was quantified using the relative quantification method by normalization with reference gene [47]. Statistical analysis using the bestkeeper tool was used to compare and select the best reference gene with stable expression across the human adrenal samples [48]. Reference genes with significantly different Ct values (*p*-value < 0.01) between the samples were excluded. Furthermore, primer efficiency and the associated correlation coefficient R2 of the selected reference gene were determined by the standard curve method in serially diluted cDNA samples [49]. In the case of miRNA reference genes, miR-16-5p [48,50,51] and RNU6B [52] previously used in similar studies were compared. MiR-16-5p was found to show the most stable expression levels across the samples with a *p*-value of 0.452 in comparison to RNU6B which had a *p*-value of 0.001. In the case of RNA reference genes, PPIA [53] and GAPDH [54] were compared. Here, PPIA was found to show the most stable expression levels across the samples with a *p*-value of 0.019 in comparison to GAPDH which had a *p*-value of 0.003. Therefore, these genes were used for the normalization of miRNA and RNA expression in human adrenal samples.

### 4.4. Target Screening

In silico prediction of the possible miRNA targets was performed using the miRNA target database, TargetScan, and miRTarBase [19,37]. The top predicted targets were further screened based on their expression status in the RNA-seq data from the adrenocortical tissues of CPA, PBMAH, and controls (unpublished data). Pathway analyses of the targets were performed using Reactome [55] and Panther [56] online platforms. The selected downregulated targets were analyzed by QPCR in the adrenocortical samples to confirm their expression status. The successfully validated candidates were then analyzed for regulation by the miRNA using a dual luciferase assay [57].

### 4.5. Dual Luciferase Assay

The interaction between the predicted 3′-UTR region of Cyb5a and miR-1247-5p was detected using a luciferase activity assay. The 3′UTR sequences of Cyb5a (129 bp) containing the predicted miR-1247-5p binding sites (psiCHECK-2 Cyb5a 3′UTR WT) were cloned into the psiCHECK-2 vector (Promega, Fitchburg, WI, USA). A QuikChange Site-Directed Mutagenesis kit (Agilent Technologies, CA, USA) was used to mutate the miR-1247-5p binding site (psiCHECK-2 Cyb5a 3′UTR mutant) according to the manufacturer’s protocol. All the sequences were verified by Sanger sequencing. Then, 200 ng of the plasmid was used for each transfection. Synthetic miR-1247-5p mimics and specific oligonucleotides that inhibit endogenous miR-1247-5p (miR-1247-5p inhibitors) were purchased from Promega and 100 nmol of the molecules were used for each transfection according to the manufacturer’s protocol. For the assay, HeLa cells were seeded in 96-well plates and incubated for 24 h. The following day, cells were transfected using the following different conditions: (1) psiCHECK-2 Cyb5a 3′UTR WT + miR-1247-5p mimic; (2) psiCHECK-2 Cyb5a 3′UTR WT + miR-1247-5p inhibitor; (3) psiCHECK-2 Cyb5a 3′UTR WT + water; (4) psiCHECK-2 Cyb5a 3′UTR mutant + miR-1247-5p mimic; (5) psiCHECK-2 Cyb5a 3′UTR mutant + miR-1247-5p inhibitor; (6) psiCHECK-2 Cyb5a 3′UTR mutant + water. Forty-eight hours later, luciferase activity in the cells was measured using the dual luciferase assay system (Promega, Fitchburg, WI, USA) in accordance with the manufacturer’s instructions. Renilla luciferase activity was normalized to firefly luciferase activity. Each treatment was performed in triplicate. Any interaction between the cloned gene, Cyb5a (WT and mutant), and miR-1247-5p mimic is accompanied by a decrease in luminescence. This decrease in luminescence would not be observed when the plasmids are transfected with the miR-1247-5p inhibitor, indicating that observed luminescence differences are caused by specific interactions between the plasmid and the miR-1247-5p mimic. Transfection of the plasmid with water corrects any background interactions between the cloned gene and endogenous miRNAs in the culture.

### 4.6. In Vivo ACTH Stimulation

Experiments were performed on 13-week-old C57BL/6 J female mice (Janvier, Le Genest-Saint-Isle, France). Mice were intraperitoneally injected with 1 mg/kg of ACTH (Sigma Aldrich, Munich, Germany) and adrenals were collected after 10, 30, and 60 min of injections. In addition, control adrenals were collected from mice at baseline conditions (0 min). Mice were killed by cervical dislocation and adrenals were isolated, snap-frozen in liquid nitrogen, and stored at −80 °C for later RNA extraction. MiR-26a was taken as a housekeeping gene in the QPCR [58]. All mice were maintained in accordance with facility guidelines on animal welfare and approved by Landesdirektion Sachsen, Chemnitz, Germany.

### 4.7. Statistical Analysis and Software

R version 3.6.1 was used for the statistical analyses. To identify RNAs differentially expressed, a generalized linear model (GLM, a flexible generalization of ordinary linear regression that allows for variables that have distribution patterns other than a normal distribution) in the software package edgeR (Empirical Analysis of DGE in R) was employed to calculate *p*-values [45,59]. *p*-values were adjusted using the Benjamin–Hochberg false discovery rate (FDR) procedure [60]. Disease groups were compared using the unpaired Mann–Whitney test, and to decrease the false discovery rate a corrected *p*-value was calculated using the Benjamin–Hochberg method. *p* adjusted < 0.05 and log2 fold change >1.25 was applied as the cut-off for significance for NGS data. GraphPad Prism Version 8 was used for the statistical analysis of QPCR. To calculate differential gene expression, the dCt method (delta Ct (cycle threshold) value equals target miRNA’s Ct minus housekeeping miRNA’s Ct) was used (Microsoft Excel 2016, Microsoft, Redmond, WA, USA). For QPCR, an ANOVA test with Bonferroni correction was used [61] to assess significance; *p*-values < 0.05 were considered significant.

## 5. Conclusions

In conclusion, while comprehensive information regarding the role of miRNAs in acute and chronic phases of steroidogenesis is available, there is little known about the pathological independent role of miRNAs in the pathology of CS. In our study, we have described ACTH-independent miR-1247-5p and miR-379-5p expression in CS for the first time. Thus, by regulating different genes in the WNT signaling pathway, the miRNAs may individually contribute to the Cushing’s pathology in specific subtypes.

## Figures and Tables

**Table 1:** Analysis of the predicted targets of miR-1247-5p and their expression levels in comparison to controls (log2 fold change). Published literature on target genes in reference to CS is highlighted in bold.

Predicted Targets	Name	RNA-Seq CPA vs. Controls
ITIH5	Inter-alpha-trypsin Inhibitor Heavy Chain Family, Member 5	−3.42
**GABBR2** **PMID: 31581124**	**Gamma-aminobutyric Acid (GABA) B Receptor, 2**	**−3.32**
C14orf180	Chromosome 14 Open Reading Frame 180	−2.31
TTC36	Tetratricopeptide Repeat Domain 36	−2.16
SEMA6D	Semaphorin 6D	−1.52
**CYB5A** **PMID: 31581124**	**Cytochrome B5 Type A (Microsomal)**	**−1.26**
FUT1	Fucosyltransferase 1	−0.95
**GNAQ** **PMID: 31581124**	**Guanine Nucleotide Binding Protein (G Protein), Q Polypeptide**	**−0.51**

**Table 2:** Clinical characteristics of the patient groups. Data are given as median with 25th and 75th percentiles in brackets. CPA, cortisol producing adenoma; CD, Cushing’s disease.

	n	Age at Surgery (Years)	Sex (%Female)	Baseline ACTH (pmol/L)	Cortisol 24 h Urine (nmol/Day)	Midnight Salivary Cortisol (nmol/L)	Cortisol after 1 mg Dexamethasone (nmol/L)
CPA	7	52 [40; 57]	62	0.4 [0.4; 0.8]	1684 [1449; 2120]	16.0 [12.7; 24.3]	386 [326; 431]
CD	8	42 [37; 52]	75	10.5 [7.8; 12.1]	2211 [1949; 2339]	21.1 [12.0; 27.3]	373 [233; 622]
PBMAH	10	61 [59; 68]	100	0.6 [0.4; 0.7]	994 [619; 1256]	15.6 [9.3; 21.1]	250 [206; 481]
Ectopic	3	69 [62; 71]	66	51.3 [35.0; 68.8]	1168 [939; 22,771]	95.2 [54.8; 538.9]	1169 [884; 1454]
Controls	8	53 [48; 58]	50	NA	NA	NA	NA

## Data Availability

The miRNA-seq data generated in this study have been submitted to the NCBI (PRJNA847385).

## Supplementary Materials

The following supporting information can be downloaded at: https://www.mdpi.com/article/10.3390/ijms23147676/s1.
