# Supplementary material for: Characterization of Adrenal miRNA-Based Dysregulations in Cushing’s Syndrome
Source: Int J Mol Sci. 2022 Jul 12;23(14):7676. doi: 10.3390/ijms23147676 (PMC9320303; doi:10.3390/ijms23147676)
Supplement: Supplementary file 1 [file ijms-23-07676-s001.zip › ijms-1768658-supplementary.pdf]

Figure S1: Principal component analyses of the samples used in sequencing.

The number of samples used for the sequencing was 24. controls, n=8. CD, n=8. Among the 7 CPA samples, 3 of them were positive for PRKACA mutation. However, no significant clustering was found between the samples based on the sequencing profile alone.

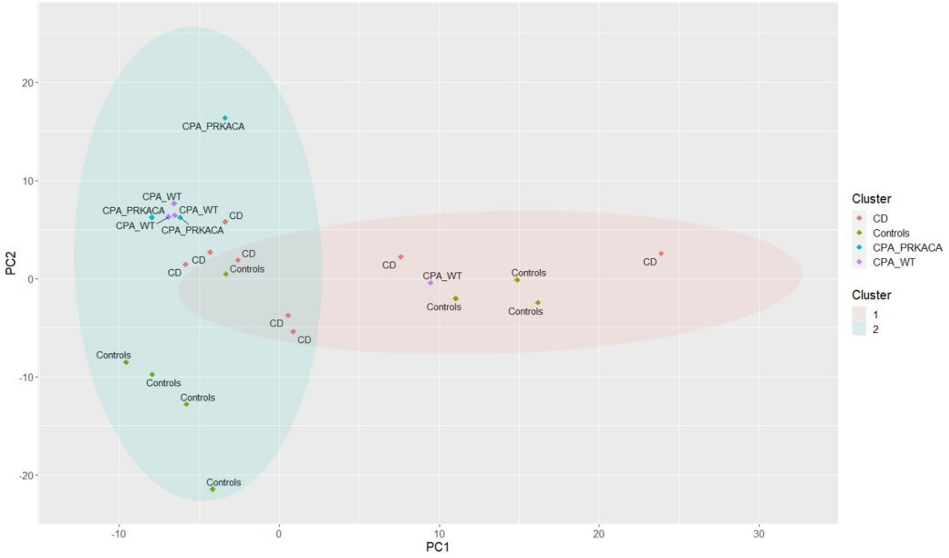

Figure S2: Expression status of the target genes in RNA seq data.

The target genes related to WNT signaling pathways from the in silico analyses were shortlisted. Their expression status from the RNA seq data was determined and represented here. Data are represented as Mean ± Standard Error of Mean (SEM) of log2fold change values. Statistics: ANOVA test with Bonferroni correction to detect significant differences between patient groups with at least a signification of p value <0.05 (*).

Fig.S2

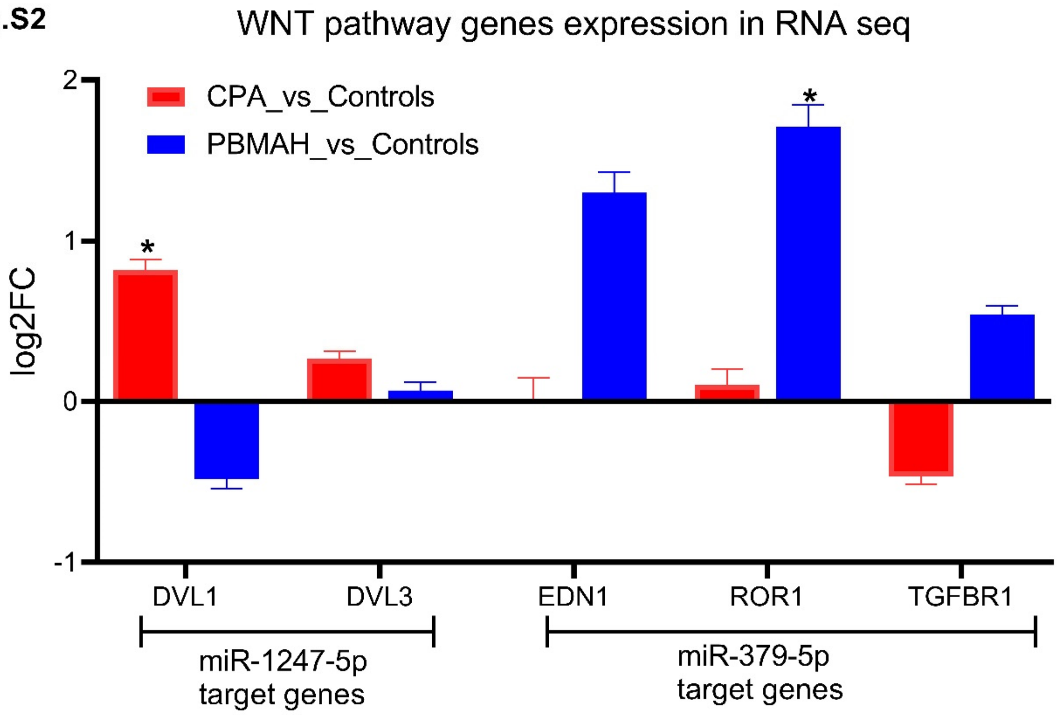

Table S1:

Comparison of the log2Fold-change levels of selected miRNAs in miRNA-seq and QPCR.miRNAs with concordant data in sequencing and QPCR are highlighted in bold. *P<0.05, **P<0.01, and ***P<0.001

	RNA sequencing			QPCR		
miRNA	CPA vs Controls	CD vs Controls	CPA vs CD	CPA vs Controls	CD vs Controls	CPA vs CD
**miR-139-3p**	**2.94*** **	**0.39**	**2.54* **	**1.44* **	**-0.89**	**-2.33*** **
miR-146b-5p	1.52*	0.68	0.84	0.26	0.29	0.04
**miR-150-5p**	**1.26**	**-0.84**	**2.10* **	**0.75* **	**-1.41**	**-2.17*** **
miR-379-5p	1.65***	0.37	1.28*	0.88	1.90**	1.02
miR-503-5p	2.12**	0.05	2.08*	0.87	0.80	-0.07
**miR-1247-5p**	**3.25*** **	**0.90**	**2.35* **	**2.49*** **	**0.00**	**-2.48*** **
**miR-144-5p**	**-2.12** **	**-1.83* **	**-0.30**	**-0.97**	**1.96** **	**2.92*** **
**miR-144-3p**	**-2.51*** **	**-2.41** **	**-0.10**	**-1.33**	**-1.64** **	**-0.31**
miR-363-3p	-1.84**	-1.70*	-0.13	-1.08	-1.20	-0.12
miR-486-5p	-1.58*	-1.57*	-0.01	-0.77	-1.31	-0.54
miR-551b-5p	-5.17**	-0.39*	0.00	-1.54	2.15	3.69
